# Regional and temporal trends in malaria commodity costs: an analysis of Global Fund data for 79 countries

**DOI:** 10.1186/1475-2875-12-466

**Published:** 2013-12-30

**Authors:** Francis Wafula, Ambrose Agweyu, Kate Macintyre

**Affiliations:** 1 Aidspan, P. O. Box 66869–00800, Nairobi, Kenya; 2 KEMRI/Wellcome Trust Research Programme, Nairobi, Kenya

**Keywords:** Global Fund, Malaria commodities, Procurement

## Abstract

**Background:**

Although procurement consumes nearly 40% of Global Fund’s money, no analyses have been published to show how costs vary across regions and time. This paper presents an analysis of malaria-related commodity procurement data from 79 countries, as reported through the Global Fund’s price and quality reporting (PQR) system for the 2005–2012 period.

**Methods:**

Data were analysed for the three most widely procured commodities for prevention, diagnosis and treatment of malaria. These were long-lasting insecticide-treated nets (LLINs), malaria rapid diagnostic tests (RDTs) and the artemether/lumefantrine (AL) combination treatment. Costs were compared across time (2005–2012), regions, and between individual procurement reported through the PQR and pooled procurement reported through the Global Fund’s voluntary pooled procurement (VPP) system. All costs were adjusted for inflation and reported in US dollars.

**Results:**

The data included 1,514 entries reported from 79 countries over seven years. Of these, 492 entries were for LLINs, 330 for RDTs and 692 for AL. Considerable variations were seen by commodity, although none showed an increase in cost. The costs for LLINs, RDTs and AL all dropped significantly over the period of analysis. Regional variations were also seen, with the cost for all three commodities showing significant variations. The median cost for a single LLIN ranged from USD 4.3 in East Asia to USD 5.0 in West and Central Africa. The cost of a single RDT was lowest in West and Central Africa at US$ 0.57, and highest in the Latin American region at US$ 1.1. AL had the narrowest margin of between US$ 0.06 per tablet in sub-Saharan Africa and South Asia, and US$ 0.08 in the Latin American and Eastern Europe regions.

**Conclusion:**

This paper concludes that global procurement costs do vary by region and have reduced overall over time. This suggests a mature market is operating when viewed from the global level, but regional variation needs further attention. Such analyses should be done more often to identify and correct market insufficiencies.

## Background

Malaria infected 219 million people leading to 660,000 deaths in 2010 [1]. The numbers have reduced gradually over the past decade. It is estimated, for instance, that malaria deaths have reduced by a third in sub-Saharan Africa over the period [1]. These successes can be attributed to the combined efforts of governments, civil society and private sector, with support from bilateral and multilateral partners such as the Global Fund to fight AIDS, TB and Malaria (the Global Fund), the US President’s Malaria Initiative (PMI) and the Roll Back Malaria partnership. The Global Fund remains the largest funder for malaria, contributing nearly half of all international financing for the disease [2]. Between 2002–2012, Global Fund financing helped provide over 310 million insecticide-treated nets for malaria control [2]. It is estimated that more than 260 million malaria cases were treated over the same period.

Nearly 40% of Global Fund money disbursed to countries goes towards procurement, making it a major player in the market for commodities. To ensure the money is used efficiently, the Global Fund introduced a price reporting mechanism, the Price and Quality Reporting (PQR) system, in 2005 [3], and a voluntary pooled procurement (VPP) system in 2009 [4].

The PQR is a web-based system that collects procurement data for anti-malarials, bed nets and malaria rapid diagnostic tests (RDTs), as well as selected HIV and TB commodities. Grant recipients enter procurement data on delivery of each consignment, with non-compliers facing the risk of getting a poor grant rating, and lower subsequent disbursements [5,6]. The VPP, on the other hand, was designed to lower costs through pooled purchasing. Under the system, VPP agents place orders on behalf of countries, and report procurement information directly to the Global Fund. VPP procurement information is not entered into the PQR system.

While the PQR has collected large amounts of data, little analyses have been published to show how procurement costs vary by region and time. Past medicine price analyses have focused on comparing prices paid by consumers across countries (country-level comparison) rather than comparing procurement costs across countries and regions (global-level comparison). This paper describes procurement cost trends for selected malaria commodities over the seven-year period.

## Methods

Three Global Fund datasets were used: one containing PQR- data showing commodity costs for individual country procurement, the second reporting data for procurements done through the VPP system, and the final one containing information on the location (region) of grant recipients.

The Global Fund provides grants across 8 regions: East Asia and the Pacific (EAP), Eastern Europe and Central Asia (EECA), Latin America and the Caribbean (LAC), Middle East and North Africa (MENA), South Asia, sub-Saharan Africa -East Africa (SSA-EA), sub-Saharan Africa (SSA-SA) and sub-Saharan Africa- West and central Africa (SSA-WCA).

Three commodities were subsequently selected for analysis: long-lasting insecticide-treated nets (LLINs), RDTs and artemether/lumefantrine (120 mg/20 mg). The three were the most commonly procured commodities for prevention, diagnosis and treatment of malaria respectively.

Initial inspection revealed data outliers, necessitating the use of medians and interquartile ranges (IQRs), rather than means. Past analyses of commodity prices have also used medians [7]. All costs were reported in USD, and were adjusted for inflation using World Bank provided values [8]. Extreme values deemed to arise from data entry errors were omitted from the analyses (omitted values were less than 1% of the analysed data).

Unit costs calculated were the cost of a single tablet for AL; the cost of a bed net and the cost of a single RDT. Information on RDT brands was unavailable, meaning unit cost calculations could not be done for kits with the same exact specifications. Caution should, therefore, be exercised when interpreting the results for this commodity. Scatter plots of unit costs were plotted against time (2005–2012), and corresponding regression coefficients and p-values reported. A linear regression line of the unit cost against time was superimposed on the scatter plots to illustrate the trend. Regional median prices were calculated and presented in tables, with corresponding IQR values, and p-values showing the level of significance of the regional variations.

Finally, PQR reported costs were compared to those reported through the VPP. We compared the regression coefficients of PQR and VPP costs for the three commodities to test the null hypothesis Ho: Coefp = Coefv, where Coefp is the regression coefficient for PQR costs, and Coefv is the regression coefficient for VPP costs. A model was then fitted including the variables PQR, time and an interaction term representing the product of PQR and time. The interaction term PQR-time was used to test the null hypothesis, and findings discussed. Analyses were done using STATA version 12 (Stata Corp, Texas, USA).

## Results

There were 1,514 entries from 79 countries spread across the eight Global Fund regions. Of the 1,514 entries, 492 were for LLINs, 330 for RDTs and 692 for the AL (20 mg + 120 mg) combination. These entries included both PQR and VPP reported data. There was an overall annual increase in number of purchases reported through the PQR, with the 2003 – 2005 period having less than 100 entries, and the period after 2011 having more than 400 entries per year.

### Comparing median costs by Global Fund region

The median cost for LLINs ranged from USD 4.3 in East Asia to USD 5.0 in the SSA-WCA region (Table 1). For RDTs, median costs were lowest in SSA-WCA 0.57, and highest in the LAC region (USD 1.1, IQR 0.82-1.6). The EECA region had only one purchase reported over the period of analysis. However, brand details were not provided, making it impossible to understand how unit costs varied for malaria RDTs with similar specifications. The cost of AL also showed variations across the eight regions, with the highest unit costs being reported in the LAC and EECA regions (both USD 0.08). The Kruskal-Wallis test showed that the regional median cost variations were significant at the 95% level across all three commodities (Table 1).

**Table 1:** Median and inter-quartile costs (2005–2012) for commodities by GF region

**Global fund region**	**Median costs (number of purchases) (inter-quartile ranges) for the 2005–2012 period**		
	**LLINs**	**Malaria test kits**	**AL anti-malarial**
East Asia/Pacific	4.3 (92) (3.9-5.2)	0.63 (59) (0.55-0.76)	0.07 (115) (0.06-0.07)
Eastern Europe/Central Asia	6.1 (19) (5.5-7.1)	0.80 (1) (0.80-0.80)	0.08 (5) (0.06-0.08)
Latin America/Caribbean	5.2 (32) (4.0-6.0)	1.1 (29) (0.82-1.6)	0.08 (20) (0.06-0.09)
North Africa/Middle East	4.8 (59) (3.5-6.1)	0.64 (22) (0.56-0.81)	0.07 (24) (0.06-0.07)
South Asia	5.2 (59) (4.6-5.7)	0.61 (59) (0.32-0.83)	0.06 (27) (0.06-0.08)
SSA: East Africa	6.0 (95) (4.8-7.8)	0.87 (72) (0.56-1.1)	0.06 (206) (0.06-0.07)
SSA: Southern Africa	5.5 (20) (5.1-6.2)	0.68 (23) (0.55-0.77)	0.06 (81) (0.06-0.07)
SSA: West and Central Africa	5.5 (80) (4.5-6.6)	0.57 (57) (0.46-0.97)	0.06 (142) (0.06-0.07)
**p- Value (Kruskal-Wallis Test)**	**p < 0.001**	**p < 0.001**	**p = 0.001**

### Procurement cost trends over the period of analysis (2005–2012)

**Figure 1:**
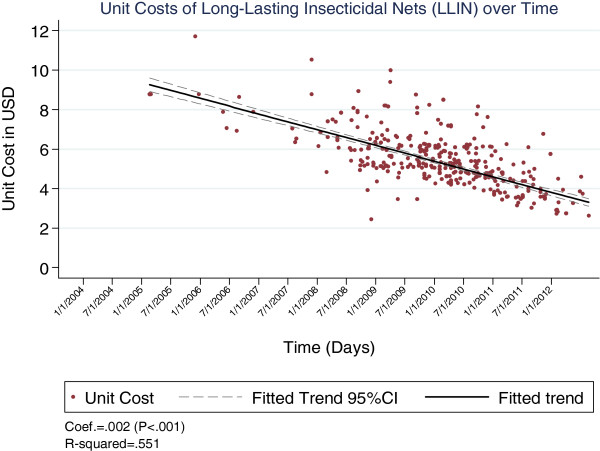
Unit costs of LLINs over time.

**Figure 2:**
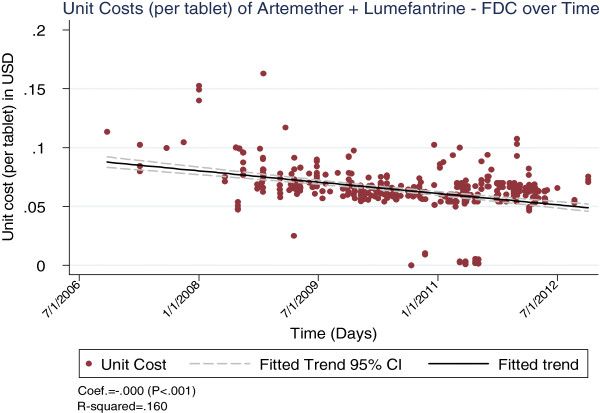
Unit costs of the artemether/lumefantrine combination over time.

**Figure 3:**
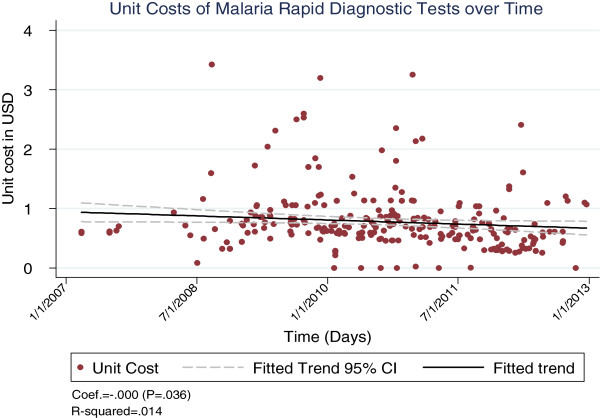
Unit costs of malaria RDTs over time.

The cost trends varied across commodities, with LLINs and AL showing significantly large reductions over the period (p < 0.001 for both, see Figures 1 and 2), while RDTs showed a relatively lower but still significant decline over the period (p = 0.04, see Figure 3).

### Comparing cost trends for VPP and non-VPP procurement

**Figure 4:**
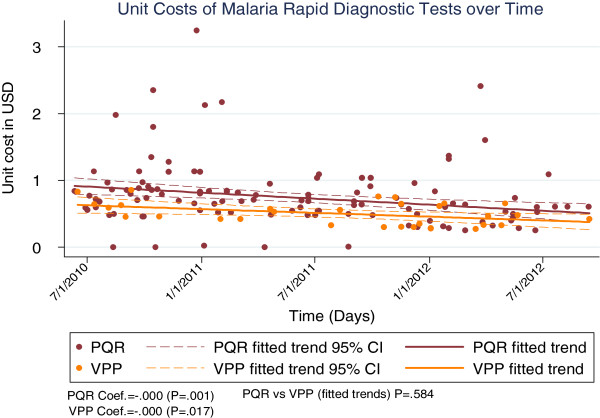
Comparing costs of direct and VPP procurement of RDTs.

**Figure 5:**
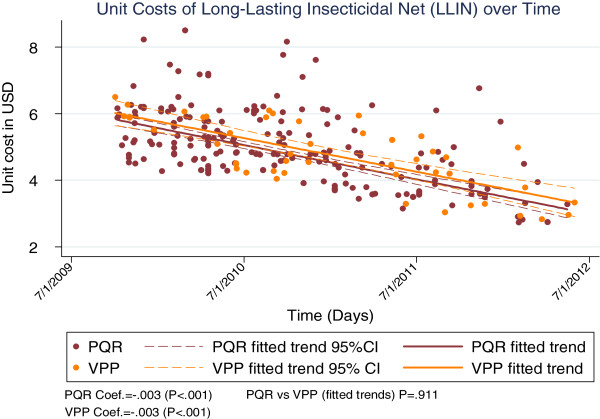
Comparing costs of direct and VPP procurement of LLINs.

**Figure 6:**
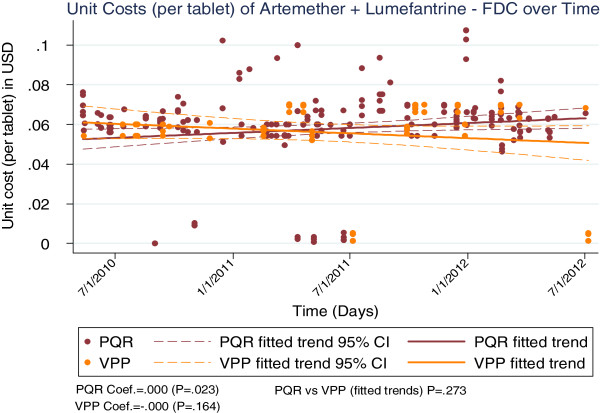
Comparing costs of direct and VPP procurement of AL.

Analyses for VPP costs were only done for the period the VPP was in existence (period VPP data were available, 2009–2012). For this period, both the PQR and VPP costs showed significant declines for RDTs (p = 0.001 and p = 0.02 for PQR and VPP respectively, Figure 4) and LLINs (p < 0.001 for both PQR and VPP, Figure 5). However, findings were mixed for AL, with PQR costs showing a significant decline (p = 0.02) while the change in VPP costs was not significant at the 95% level (p = 0.16, Figure 6). Comparing PQR and VPP trends showed that the differences were not statistically significant across all three commodities (p = 0.9, p = 0.3 and p = 0.6 for LLINs, AL and RDTs respectively, see Figures 4, 5, and 6).

## Discussion

In a well functioning market, prices for commodities fall as newer and more sophisticated ones enter the market. However, health care markets are well known to have higher risks of monopolization and information flow inadequacies, both of which may lead to reduced supply and increased prices [9,10]. For this reason, prices for health care commodities should be monitored continuously, and corrections made where market failures emerge. This work contributes to the objective by providing information on regional and temporal trends in the procurement costs for three key malaria commodities.

The analysis found significant declines in cost trends over the seven years, with LLINs and AL showing larger declines than RDTs. The drop in the cost for LLINs possibly reflects a rise in competition and demand that followed an overall increase in malaria funding over the period. According to WHO, international funding for malaria increased from below US$ 100 million in 2000 to US$ 1.71 billion in 2010 [1]. Domestic funding was also reported to have increase in most countries over the period.

Low LLIN prices are not however a direct reflection of cost-efficiency. Indeed, malaria control programs are encouraged to consider longevity alongside cost [11]. Higher initial costs may reduce the cost-per-year and result in more savings if the nets have a considerably longer lifespan [11,12]. The Global Fund has been criticized for overemphasizing low unit cost at the expense of longevity (although the Global Fund promotes procurement of the lowest priced WHO-prequalified LLINs, longevity of prequalified nets also varies) [11]. Future analyses should look at the price benefits of procuring LLINs of varying durability across different settings. This could not be done because brand information was not provided.

The cost of AL also showed a significant decline over the period of analysis. This may be attributed mainly to a decade-long agreement between the Global Fund and Novartis, a major producer of AL. The agreement stipulated that Novartis would supply AL to public sector buyers at cost price [13]. Several rounds of price negotiations between Novartis and the Global Fund over the period resulted in further declines in AL prices, which is reflected in the observed trends. These negotiations explain why AL procurement costs did not increase in the face of a volatile market for the artemisinin raw material over the period of analysis [14].

Another factor that may have contributed to the AL procurement cost decline was the overall growth in the market for ACTs. The number of WHO-prequalified ACT manufacturers rose from one manufacturer producing a single product in 2005, to seven firms producing at least 11 different ACT formulations by 2011 [15]. Growth in demand was also seen, with the number of ACT doses purchased by donors increasing from 11.2 million to 217 million between 2005 and 2010 [15]. Costs are, however, unlikely to drop much further, as production processes for ACT are believed to be optimum [16]. Further cost reductions may require the introduction of new combination molecules.

Unlike LLINs and AL, the cost for RDTs only showed a mild decline over the period. This result must, however, be interpreted with caution, as information on brand names was unavailable. Unlike the other two commodities, RDTs vary considerably, from simple kits designed to test for *Plasmodium falciparum* only, to more sophisticated brands that can detect *P. falciparum* and *Plasmodium vivax*[17]. Not having this information meant inference could not be made on whether procured kits were of the same exact specifications.

The slight decline in the overall RDT costs is nonetheless positive, especially considering the growing importance of diagnostic confirmation. RDTs have been linked to better treatment and reduced wastage of medicines [18]. However, while demand for AL is expected to fall with the shrinking malaria map, demand for RDTs is likely to remain high, particularly in areas where fevers are common [16]. This underscores the importance of pursuing even lower prices for RDTs.

There were significant regional variations in the cost for all three commodities. These variations may have resulted from countries procuring commodities from different suppliers located across different countries.

Comparing PQR and VPP procurement showed pooled procurement to have some benefit for RDTs and AL, although the differences in cost were not significant at the 95% level. However, as the VPP was only introduced in 2009, analysis could only be done over a three-year period. The marginal price benefit seen for RDTs and LLINs agrees with analyses by the Global Fund, which showed VPP prices to be either at par, or slightly lower than PQR prices [4,19]. However, the significance of the price difference would only become clear after a longer period of VPP usage. It should, nonetheless, be noted that the VPP has other potential benefits besides cost reduction. These include increased transparency in procurement, improved payment terms for countries and better availability of commodities [19].

There were some limitations in the analysis. First, only one type of ACT was included in the analysis, meaning the patterns may have differed if another combination had been used. The decision to only include the most procured ACT (AL) was informed by the need to avoid introducing additional variability that may come with combining products of different composition under one analysis. However, future analyses should consider looking at other ACT combinations, as well as differentiating between LLINs and RDTs of different quality.

Another major limitation was the presence of some outliers believed to result from data entry errors. This is a problem that the Global Fund has recognized in the past [3]. While measures such as omitting extreme values and using medians would have minimized the outlier effect, the possibility that data entry errors may have biased the results in one direction or the other cannot be ruled out. Another possible cause of bias was low data availability, especially prior to 2008. There was no way of exploring whether transactions that were captured differed systematically from those that were not captured during the early years of the PQR. It is expected that more data will be captured as the PQR system improves over time. Already, there has been a progressive increase in data captured in the PQR system across all commodities, with less than 100 total annual entries from 2003 – 2005 rising to over 4000 per year since 2011.

When doing analysis for AL, the pack sizes were not factored in. This may have caused some slight bias, especially where countries changed their AL procurement preference over time (for instance, changing from smaller (pediatric) pack sizes to larger (adult) pack sizes or the reverse. However, this would have little public health significance, as the same AL tablets (20 mg + 120 mg) are used to treat adults and children (variations only come in the number of tablets administered).

The cost patterns discussed reflect a variety of market factors. However, the role of the Global Fund and other bilateral and multilateral health initiatives cannot be overlooked, as they have contributed substantially to increased demand, lowered procurement costs and stabilized markets. As more data become available, research should describe the variations in greater depth, including the effect of the type of procuring organization (whether government or non-government), order lead times, procurement volumes and shipment or transport costs. Future analyses should also examine the effect of using the VPP system over longer periods of time.

## Conclusions

The analysis showed the value of the PQR in assessing regional and temporal cost trends. The data can inform policy on how well global markets are working. As the Global Fund and other donors continue supporting the fight against the three diseases, it is important that every caution is taken to ensure resources are used in the best way possible. By doing such analyses, inadequacies can be identified early and appropriate strategies developed. Future analyses should describe the causes of variations in greater depth, and assess the effectiveness of the VPP system over a longer period of time.

## Competing interests

The authors declare that they have no competing interests.

## Authors’ contributions

FW and KM contributed to the conceptualization of the analysis. FW, AA and KM contributed to the analysis, interpretation of results and drafting of the manuscript. All authors read and approved the final manuscript.
